# Desmoid Tumor of the Abdominal Wall

**DOI:** 10.5334/jbsr.4188

**Published:** 2026-02-09

**Authors:** Matthieu Van Herck, Filip Vanhoenacker, Charlotte Vanhoenacker

**Affiliations:** 1 Department of Radiology, Ghent University Hospital, Ghent University, Belgium; 2 Department of Radiology, AZ Sint‑Maarten, Mechelen, Belgium; 3 Faculty of Medicine and Health Sciences, University of Antwerp and Ghent University, Belgium

**Keywords:** desmoid tumor, desmoid‑type fibromatosis, aggressive fibromatosis, CT, MRI

## Abstract

*Teaching point:* Desmoid tumors are rare locally aggressive soft tissue tumors that can occur anywhere in the body and have a varying imaging appearance.

## Case History

A 35‑year‑old woman presented with abdominal pain. Palpation indicated the presence of a mass in the right upper quadrant, which was slightly tender to touch.

Abdominal CT showed a large mass originating from the right rectus abdominis muscle (Figure 1A, arrow; Figure 1B). The mass was isodense to muscle and enhanced homogeneously. Adjacent bowels, including the transverse colon and duodenum, were displaced.

**Figure 1A:**
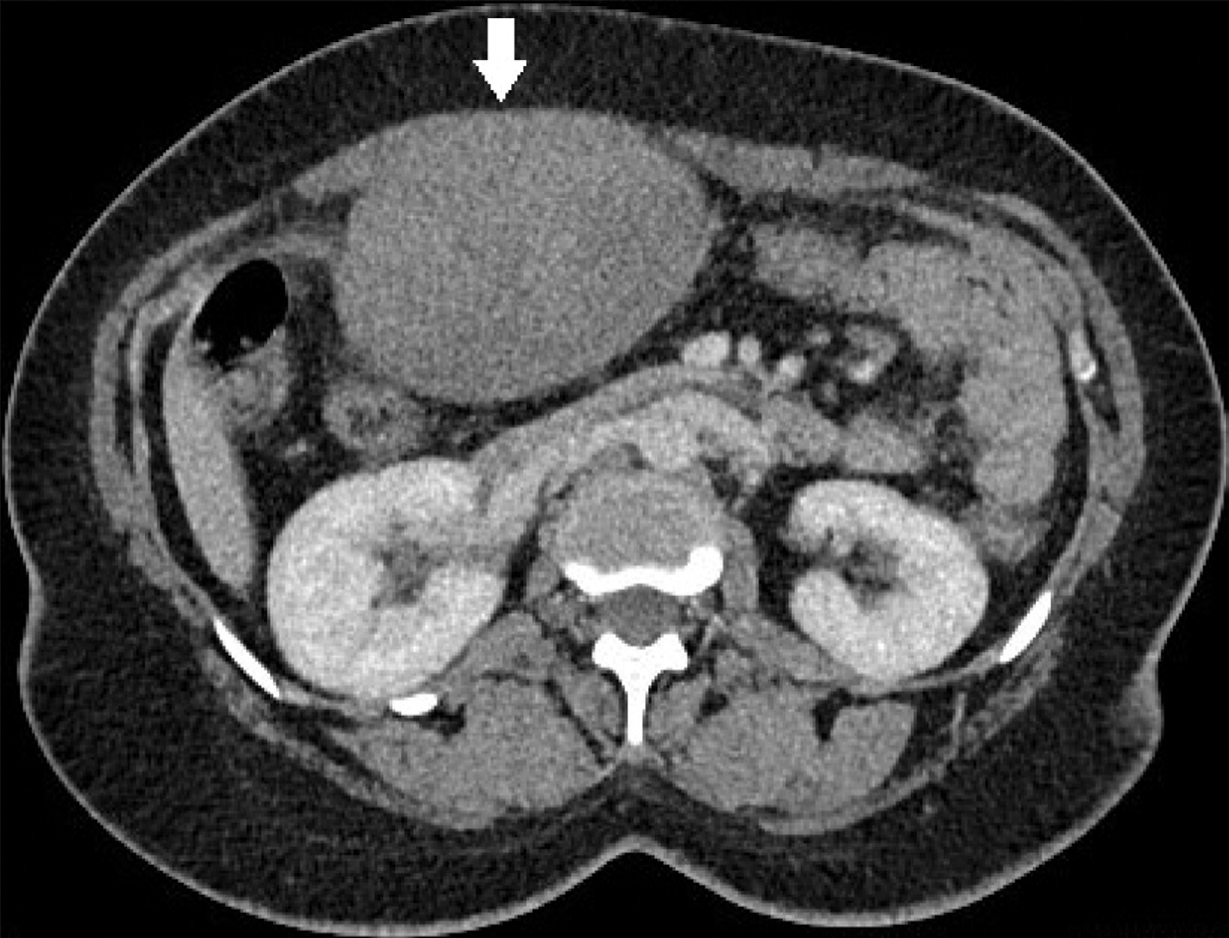
Large, well‑demarcated lesion arising from the right rectus abdominis muscle.

**Figure 1B:**
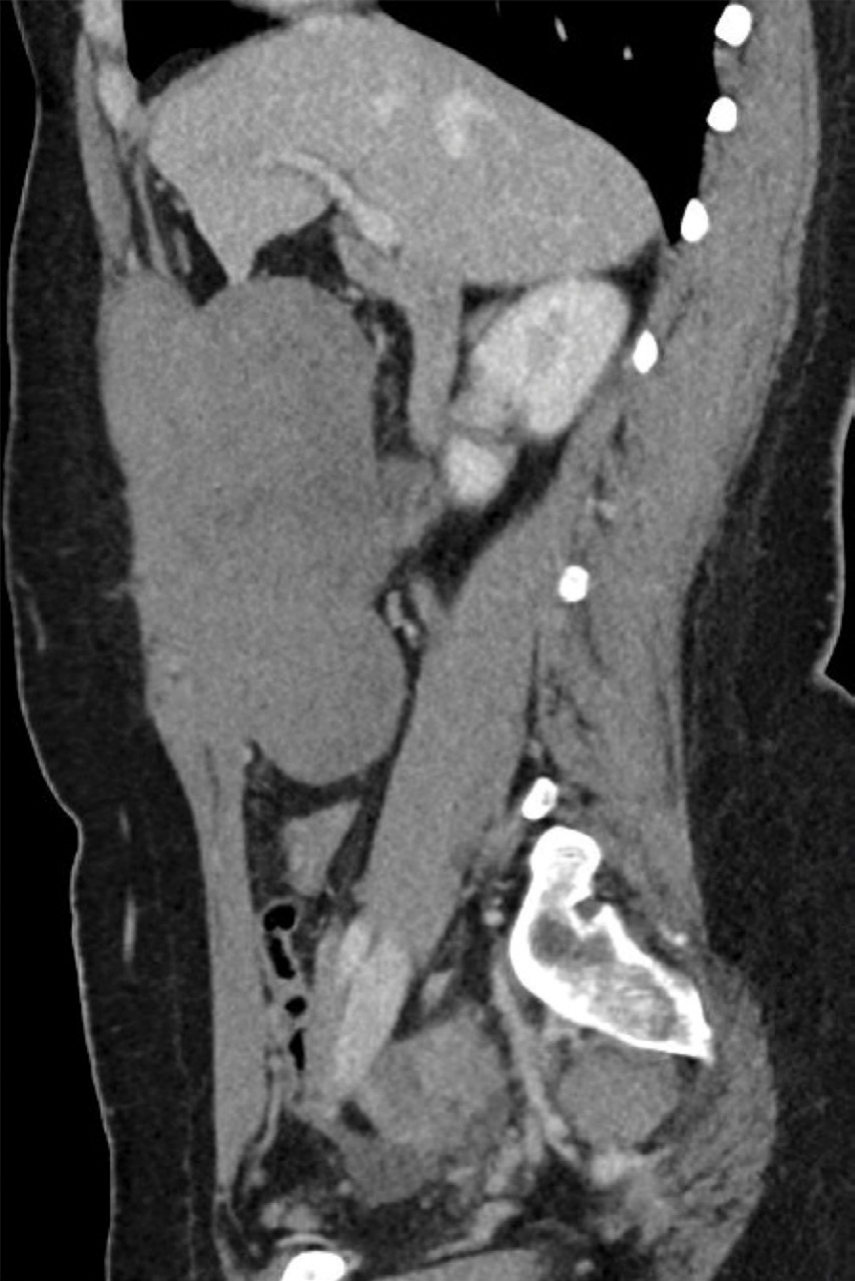
The lesion demonstrates homogeneous enhancement and quite extensive intra‑abdominal growth.

A desmoid tumor seemed the most likely diagnosis, mainly because of the location and homogeneous enhancement. The age and sex of the patient further supported this diagnosis.

**Figure 2:**
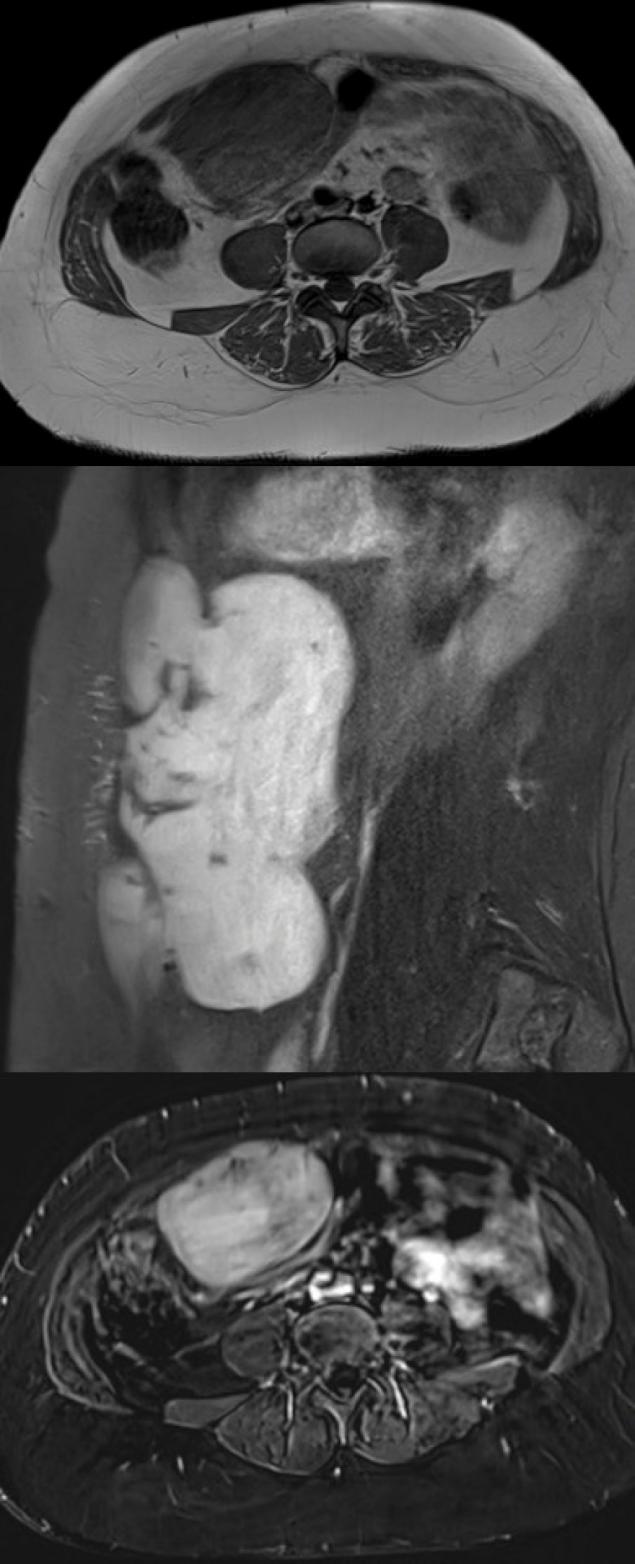
T1‑iso‑ and T2‑hyperintense lesion which enhances homogeneously, apart from a few hypointense strands.

Contrast‑enhanced MRI (Figure 2, from top to bottom: axial T1‑, sagittal FS T2‑, and axial contrast‑enhanced FS‑T1‑weighted image (WI)) shows a T1‑isointense and T2‑hyperintense lesion with homogeneous enhancement, apart from a few hypointense intralesional strands.

The lesion was biopsied under ultrasound guidance, with histopathological analysis confirming the diagnosis of a desmoid tumor (with CTNNB1 mutation). Familial adenomatous polyposis (FAP) was ruled out by colonoscopy. Follow‑up MRI six months later showed no significant evolution.

## Comments

Desmoid tumors (DTs), also known as desmoid‑type fibromatosis or aggressive fibromatosis, are rare and locally aggressive fibroblastic neoplasms, which are most frequently diagnosed between 15 and 60 years of age. Risk factors include previous trauma or surgery, pregnancy, and the use of oral contraceptives. The latter two may explain the female predominance (female‑to‑male ratio of 2:1) and the increased aggressiveness in younger patients [1].

DTs can occur anywhere in the body. They are usually classified in three groups: extra‑abdominal DTs, intra‑abdominal DTs, and abdominal wall DTs. Abdominal wall DTs are the most frequent pregnancy‑related DTs and can occur both during pregnancy and the first year following childbirth. Abdominal wall DTs can also be associated with FAP [1].

Ultrasound shows an oval, solid mass with variable echogenicity in which there can be alternating layers of hypo‑ (matrix and collagen) and hyperechogenicity (cells). Color Doppler signal can also be variable. DTs can show a ‘fascial tail sign’ because they grow along the fascia [1].

On MRI, DTs are isointense to muscle on T1‑WI and iso‑ to hyperintense to skeletal muscle on T2‑WI. Dense collagen and hypocellularity cause decreased T2 signal, whereas high cellularity causes increased T2 signal. A typical (but non‑pathognomonic) feature of DT is the dense collagenous stroma, which causes bands of low signal intensity on both T1‑WI and T2‑WI. Enhancement is variable, but is usually moderate‑to‑marked [1].

In young women, abdominal wall DTs should be differentiated from endometriosis. MRI is the most useful for differentiating, as abdominal endometriosis can cause T1‑hyperintense foci due to the presence of blood [1].
